# Paeonol Interferes With Quorum-Sensing in *Pseudomonas aeruginosa* and Modulates Inflammatory Responses *In Vitro* and *In Vivo*

**DOI:** 10.3389/fimmu.2022.896874

**Published:** 2022-05-24

**Authors:** Huaqiao Tang, Dan Yang, Ling Zhu, Fei Shi, Gang Ye, Hongrui Guo, Huidan Deng, Ling Zhao, Zhiwen Xu, Yinglun Li

**Affiliations:** College of Veterinary Medicine, Sichuan Agricultural University, Chengdu, China

**Keywords:** paeonol, *P. aeruginosa*, RAW264.7, quorum sensing, inflammation

## Abstract

Developing quorum-sensing (QS) based anti-infection drugs is one of the most powerful strategies to combat multidrug-resistant bacteria. Paeonol has been proven to attenuate the QS-controlled virulence factors of *P. aeruginosa* by down-regulating the transcription of QS signal molecules. This research aimed to assess the anti-virulence activity and mechanism of paeonol against *P. aeruginosa* infection *in vitro* and *in vivo*. In this study, paeonol was found to reduce the adhesion and invasion of *P.aeruginosa* to macrophages and resist the cytotoxicity induced by *P.aeruginosa*. Paeonol reduced the expression of virulence factors of *P.aeruginosa* by inhibiting QS, thereby reducing the LDH release and damage of *P.aeruginosa*-infected macrophages. Paeonol can inhibit bacterial virulence and enhance the ability of macrophages to clear *P.aeruginosa*. In addition, paeonol exerts anti-inflammatory activity by reducing the expression of inflammatory cytokines and increasing the production of anti-inflammatory cytokines. Paeonol treatment significantly inhibited the activation of TLR4/MyD88/NF-κB signaling pathway and decreased the inflammation response of *P. aeruginosa*-infected macrophages. Paeonol also significantly reduced the ability of *P.aeruginosa* to infect mice and reduced the inflammatory response. These data suggest that paeonol can inhibit the virulence of *P.aeruginosa* and decrease the inflammation response in *P.aeruginosa*-infected macrophages and mice, which can decrease the damage induced by *P.aeruginosa* infection and enhance the ability of macrophages to clear bacteria. This study supports the further development of new potential anti-infective drugs based on inhibition of QS and virulence factors.

## Introduction

*Pseudomonas aeruginosa* (*P. aeruginosa*) is a common gram-negative bacterial opportunistic pathogen that is related to the development of acute and chronic pulmonary infections in cystic fibrosis (CF), non-CF bronchiectasis, and chronic obstructive pulmonary disease with high morbidity and mortality (1–3). Lung infections caused by *P. aeruginosa* are exacerbated by the intracellular invasion and persistence of the pathogen, especially in CF patients. Intracellular invasion is a favorable mechanism for pathogens to avoid host defense and antimicrobial therapy (4). The intrinsic ability for *P. aeruginosa* to invade and adhere to host cells of infection is ascribed to its impenetrable biofilm formation and an elaborate arsenal of virulence factors designed to damage the host membrane barrier and evade immune responses, coupled with its rapidly acquired antibiotic resistance (5, 6).

Quorum-sensing (QS) is a signaling mechanism by which cell-to-cell communication receives input from adjacent cells and coordinates collective behavior, enabling the bacteria to survive within the host, releasing a range of virulence factors, and developing biofilm (7, 8). *P. aeruginosa* produces an armory of factors associated with intracellular communication and extracellular virulence, regulated by the quorum-sensing system of the diffusible signaling molecule (N-acyl L-homoserine lactone, AHLs) and determines the pathogenesis of bacteria (9, 10). Early colonization and dissemination of *P. aeruginosa* infection in host tissues is initiated by proteases and elastases, while pyocyanin interferes with various cellular functions such as chelated iron absorption, respiration, and enhances virulence expression (11, 12). Rhamnolipid promotes the surface movement of *P. aeruginosa* to form biofilms and participates in the diffusion of mature biofilms (13). *P. aeruginosa* produces different secondary metabolites and multiple virulence factors to modify host defense response, and macrophages play a crucial role in infections of patients with CF (14).

The lung innate immune response plays an important role in clearing lung pathogens. During the initial stages of *P. aeruginosa* invasion, the secretion of cytokines and chemokines induces many neutrophils to migrate to the infected site. However, the overactivation of innate immune cells (alveolar macrophages or neutrophils) can produce various proinflammatory molecules, causing severe airway injury and damaged lung function (15–17). After specifically recognizing microbial ligands, Toll-like receptors (TLRs) recruit various binding molecules to activate inflammatory gene transcription, and immune responses (18, 19). *P. aeruginosa*-induced activation of macrophages is majorly based on the recognition of pathogens by molecular pattern receptors, including TLRs, such as TLR2 and TLR4, and the mannose receptor, thus activating downstream nuclear factor kappa B (NF-κB) and mitogen-activated protein kinase (MAPK) signaling pathway increasing macrophages release of various cytokines and chemokines from response to *P. aeruginosa* infection (20–22).

Natural products targeted specific genes that inhibit the growth or pathogenicity of bacteria and control extracellular virulence, such as EGCG, Curcumin, and Eugenol-Containing Essential Oils, which have been investigated as QS inhibitors (23–25). Paeonol is a polyphenolic compound originally extracted from *Paeonia lactiflora*, *Moutan cortex*, and *Cynanchum paniculatum*, which has been used wildly due to various medicinal benefits as an analgesic, antipyretic, and anti-inflammatory agent in traditional Chinese medicine (26, 27). Previous studies have demonstrated that paeonol attenuates quorum-sensing, inhibits virulence factors, and biofilm formation (28). Paeonol has been well-documented for revealing potential anti-inflammatory activity in macrophages (29, 30), but the possible mechanism by regulating virulence and inflammation to reduce pathological damage upon pathogenic *P. aeruginosa* infection has not yet been fully studied *in vitro* and *in vivo*.

## Materials and Methods

### Bacterial Strains and Culture Conditions

*P. aeruginosa* PAO1 (ATCC 15692) was stored in our laboratory. For all the experiments, PAO1 was grown in LB broth at 37°C with shaking, collected by centrifugation (4,000 rpm for 5 min), and diluted in the appropriate experimental medium.

### Cell Culture

RAW264.7 cells were obtained from the Cell Bank of the Chinese Academic of Sciences (Shanghai, China) and cultured in DMEM medium (HyClone, Beijing, China) with 10% fetal bovine serum (FBS, TransGen Biotech, Beijing, China) and antibiotics (100 U/ml penicillin and 100 µg/ml streptomycin) (Haimen, China). The cells incubated in a cell incubator at 37°C with humidified air and 5% CO_2_.

### Cell Viability

The viability of RAW264.7 macrophage cells was determined using the CCK8 assay. Briefly, RAW264.7 (1 × 10^5^ cells/mL) were suspended in complete cell culture media, and 150 μL of the cell suspension was cultured in 96-well plates for 24 h. After treatment with paeonol, cell viability was evaluated using the Cell Counting Kit-8 (Sangon, Biotech, Shanghai, China).

### *P. aeruginosa* Infection of Macrophages

The *P. aeruginosa*-infected macrophages model used in this study was performed as described previously (31). RAW264.7 cells were seeded into 12-well tissue culture plates at a density of 5×10^5^ cells/well and then reached 80-90% confluence. *P. aeruginosa* were collected and suspended to 10^8^ CFUs/mL in DMEM without antibiotics. RAW264.7 cells were incubated with *P. aeruginosa* at three multiplicity of infection (MOIs, 25, 50, or 100) in DMEM without antibiotics. After 2 h or 4 h of PAO1 infection, cells were washed with phosphate-buffered saline (PBS), then lysed with 0.5% Triton-X for 15 min at 37 °C. For invasion assay, extracellular bacteria were killed with 200 μg/mL gentamicin for 1 h. The attachment and invasion levels were assessed by bacterial plate count.

### Cell Treatment

At 80%–90% confluence, cells were washed third with PBS, then subsequently incubated with *P. aeruginosa* of 100 (MOI) in antibiotic-free DMEM for 4 h. Cells were divided into four groups: the first part involved the control group (RAW264.7 cells group) and the *P. aeruginosa* infection group (PAO1). On this basis, the second part was set up as three parallel groups, namely PAO1 (MOI = 100:1, infection for 4 h) as cells model group, paeonol pretreatment (32 μg/mL, 64 μg/mL and 128 μg/mL, pretreatment for 3 h) + PAO1 group and paeonol (32 μg/mL, 64 μg/mL and 128 μg/mL, treatment for 4 h) + PAO1 treatment group. These concentrations were no bacteriostatic effect, and previous studies confirmed that the MIC of paeonol against *P.aeruginosa* was greater than 512 μg/ml.

### Determine mRNA Expression Levels of QS-Related Virulence Genes of *P. aeruginosa* by qPCR

Total RNA was reverse-transcribed to cDNA, and qPCR was carried out by the instruction of commercial kit. **Table 1** shows the primers used to amplify *lasI/R, rhlI/R, pqsA/R, LasA, LasB, rhlA, rhlC, phzm, phzM, phzH, phzS*, and *rpoD* (reference gene). qPCR data were analyzed to calculate the relative differences in mRNA expression using the 2^-△△CT^ method.

**Table 1:** qPCR primers of *P. aeruginosa* for analysis of gene expression.

Genes	Primer sequences (5, -3, )	
***lasI***	F: CGCACATCTGGGAACTCA	R: CGGCACGGATCATCATCT
***lasR***	F: CTGTGGATGCTCAAGGACTAC	R: AACTGGTCTTGCCGATGG
***rhlI***	F: GTAGCGGGTTTGCGGATG	R: CGGCATCAGGTCTTCATCG
***rhlR***	F: GCCAGCGTCTTGTTCGG	R: CGGTCTGCCTGAGCCATC
***pqsA***	F: GACCGGCTGTATTCGATTC	R: GCTGAACCAGGGAAAGAAC
***pqsR***	F: CTGATCTGCCGGTAATTGG	R: ATCGACGAGGAACTGAAGA
***lasA***	F: CTGTGGATGCTCAAGGACTAC	R: AACTGGTCTTGCCGATGG
***lasB***	F: AACCGTGCGTTCTACCTGTT	R: CGGTCCAGTAGTAGCGGTTG
***rhlA***	F: TGGCCGAACATTTCAACGT	R: GATTTCCACCTCGTCGTCCTT
***rhlC***	F: GCCATCCATCTCGACGGAC	R: CGCAGGCTGTATTCGGTG
***phzM***	F: ACGGCTGTGGCGGTTTA	R: CCGTGACCGTCGCATT
***phzA***	F: AACGGTCAGCGGTACAGGGAAC	R: AACGGTCAGCGGTACAGGGAAAC
***phzH***	F: GCTCATCGACAATGCCGAACT	R: GCGGATCTCGCCGAACATCAG
***phzS***	F: CCGAAGGCAAGTCGCTGGTGA	R: GGTCCCAGTCGGCGAAGAACG
***rpoD***	F: GGGCGAAGAAGGAAATGGTC	R: CAGGTGGCGTAGGTGGAGAA

### Lactate Dehydrogenase Assay

The macrophages infected with P. aeruginosa (MOI=100:1) for 4 h, then treated with Paeonol at the concentrations of 32 μg/mL, 64 μg/mL and 128 μg/mL. The culture supernatants were harvested and centrifuged at 12,000 g for 5 min, and LDH activity was monitored by an LDH cytotoxicity assay kit (Jiancheng Biotech, Nanjing, China).

### Live/Dead cell Assay

The effects of paeonol on the viability or cytotoxicity of macrophages cells were assayed using the Calcein-AM/PI Double Stain Kit (Solarbio, Beijing, China). In brief, RAW264.7 cells were exposed to *P. aeruginosa* in DMEM without antibiotics and treated with paeonol. The cells washed with PBS three times and stained with a 100 μL Calcein-AM/PI stain solution at 37°C for 15 min. Living cells with green cytoplasmic fluorescence and dead cells with red nuclei were immediately observed by the fluorescence microscope.

### Adhesion and Invasion Assay

To further reveal the role of paeonol on macrophage-mediated phagocytosis, the intracellular killing of *P. aeruginosa*. Macrophages infected with *P. aeruginosa* (MOI=25:1 for 4 h) treated with paeonol (32, 64, and 128 μg/mL). Cells were washed with phosphate-buffered saline (PBS) and then lysed with 0.5% Triton-X for 15 min at 37°C. Extracellular bacteria were killed with 200 μg/mL gentamicin for 1 h to evaluate the intracellular bacteria. Attachment and invasion levels were assessed by bacterial plate count.

### Ultrastructure Examination by Transmission Electron Microscope

The macrophages infected with *P. aeruginosa* (MOI=25:1 for 2 h) were treated with Paeonol (128 μg/ml). The cells were washed with PBS three times, and fixed with 2.5% glutaraldehyde at 4°C overnight. Samples were collected and treated with osmium ferrocyanide for 1 h, dehydrated in graded ethanol concentrations (50%, 70%, 80%, 90%, 95%, and 100%) and 100% acetone, infiltrated, and embedded in epoxy resin. Subsequently, ultrathin sections (60-80 nm) were cut with a diamond knife on a microtome (Leica EM UC7; Leica Corporation, Germany) and were stained with saturated aqueous uranyl acetate and counterstained with 4% lead citrate, and finally observed using HT-7700 TEM (Hitachi, Tokyo, Japan).

### Flow Cytometry Assay

Paeonol (32 μg/mL, 64 μg/mL and 128 μg/mL) treated macrophages (infected with *P. aeruginosa* (MOI=25:1) for 2 h) were washed with PBS, separated to single-cell suspension, and resuspended in staining buffer (BD Biosciences). The cells were stained with F4/80 (Elabscience, E-AB-F0995C), CD86 (Elabscience, E-AB-F0994E), and CD206 (Elabscience, E-AB-F1135D) fluorescently labeled antibody, then detected by a flow cytometer (Beckman coulter, CytoFLEX) and analyzed by the Flowjo software.

### Determination of mRNA Expression Levels of Inflammation and TLR4/MyD88/NF-κB Pathway of Macrophages by qPCR

The mRNA expression levels of the TLR4/MyD88/NF-κB pathway of Paeonol treated RAW264.7 (infected with *P. aeruginosa* (MOI=100:1) for 4 h) cells were assayed by qPCR. The gene sequences of TLR4, MyD88, TRAM, NF-κB, IκB, IκBα, IKK-β, p65, p50, TNF-α, IL-1β, IL-6, IL-8, iNOS, COX-2, IL-2, IL-4, and IL-10 were retrieved from NCBI, and the primers of these genes (**Table 2**) were synthesized by HuaDa Gene company (Beijing, China). GAPDH gene was selected as the reference gene. qPCR reaction was conducted on a LightCycler^®^II real-time fluorescent quantitative PCR instrument (Roche, USA) using the PerfectStartTM Green qPCR SuperMix (TransGen Biotech, Beijing, China) following the standard steps. All data output from the qPCR experiments were analyzed using the 2^-ΔΔCT^ method.

**Table 2:** List of primers of the macrophage TLR4/MyD88/NF-κB pathway in qPCR analysis.

Genes	Primer sequences (5, -3, )	
***TNF-α***	F: CTTCTGTCTACTGAACTTCGGG	R: CAGGCTTGTCACTCGAATTTTG
***IL-1β***	F: GAAATGCCACCTTTTGACAG	R: TGGATGCTCTCATCAGGACAG
***IL-6***	F: CTTCCATCCAGTTGCCTTCT	R: CCTTCTGTGACTCCAGCTTATC
***IL-8***	F: TGTGGGAGGCTGTGTTTGTA	R: ACGAGACCAGGAGAAACAGG
***IFN-β***	F: CAGCTCCAAGAAAGGACGAAC	R: GGCAGTGTAACTCTTCTGCAT
***iNOS***	F: ACTGTGCCATCAGCAAGGTT	R: GCTCTTTGTCCATTGGGTTCTT
***COX-2***	F: TGCTGTACAAGCAGTGGCAA	R: GCAGCCATTTCCTTCTCTCC
***IL-2***	F: TGTGGAATGGCGTCTCTGTC	R: AGTTCAATGGGCAGGGTCTC
***IL-4***	F: GGTCTCAACCCCCAGCTAGT	R: GCCGATGATCTCTCTCAAGTGAT
***IL-10***	F: GCCGAGGAGATCGTCACC	R: CAGGCGTAGAAGATGTCGGA
***TLR4***	F: GGACTCTGATCATGGCACTG	R: CTGATCCATGCATTGGTAGGT
***MyD88***	F: ACTCGCAGTTTGTTGGATG	R: CACCTGTAAAGGCTTCTCG
***TRAM***	F: AGCCAGAAAGCAATAAGC	R: CAAACCCAAAGAACCAAG
***NF-κB***	F: CTGAAACTACTGATTGCTGCTGGA	R: GCTATGTGAAGAGGCGTTGTGC
***IκB***	F: GCCATCCCAGGCAGTATCTA	R: TTCCAAGACCAGACCTCCAG
***IκBα***	F: GCCCTTCTGGGATTTCCT	R: GCGGCTCCGCTTCGTTCT
***IKK-β***	F: TCAGCTAATGTCCCAGCCTTC	R: CCAGTCTAGAGTCGTGAAGC
***P65***	F: CGGGATGGCTACTATGAGGCTGACC	R: GATTCGCTGGCTAATGGCTTGCT
***P50***	F: GTGATTTGTGCCAGCCAGGAAGC	R: TTCTTAACCCGAAGCCCTTGATT
***GAPDH***	F: GGTGAAGGTCGGTGTGAACG	R: CTCGCTCCTGGAAGATGGTG

### Anti-Infection Activity of Paeonol Against *P. aeruginosa* by Quorum Sensing *In Vivo*

Fifty SPF mice (4 weeks) were obtained from Sibeifu Biotechnology Co. Ltd. (Beijing, China). The animals were housed at 22-25°C on a 12 h day-night cycle, fed standard rodent chow and sterile water ad libitum for 1 week of acclimation. All protocols for animal studies were reviewed and approved by the Animal Ethical Committee of Sichuan Agricultural University (#20210021). The mice were randomly divided into six groups (n=10): the control group, PAO 1 group, PAO 1+Pae (25 mg/kg) group, PAO 1+Pae (50 mg/kg), PAO 1+Pae (100 mg/kg), and CIP (20 mg/kg) group (positive control). The mice were administered the same dose of saline or drugs by intragastric administration for three days. In mice, infection was induced using the previously described method with modifications (32). Briefly, mice were weighed, anesthetized, and then received an intratracheal instillation of PAO 1 (2.5 × 10^8^ CFU) in 20 μl phosphate-buffered saline (PBS) to model the acute infection of PAO 1. The mice were anesthetized and sacrificed after 24 h of infection, and lung tissue samples were harvested rapidly for subsequent analysis. Then the bacterial load, the expression of virulence, and inflammation cytokines were evaluated by PCR.

### Statistical Analysis

All values are expressed as mean ± standard deviation (SD). All statistical analyses were performed using SPSS 20.0. Differences between all groups were analyzed using the one-way ANOVA test, and the result with *P* < 0.05 or *P* < 0.01 was considered statistically significant.

## Results

### The Cytotoxicity Evaluation of Paeonol

**Figure 1:**
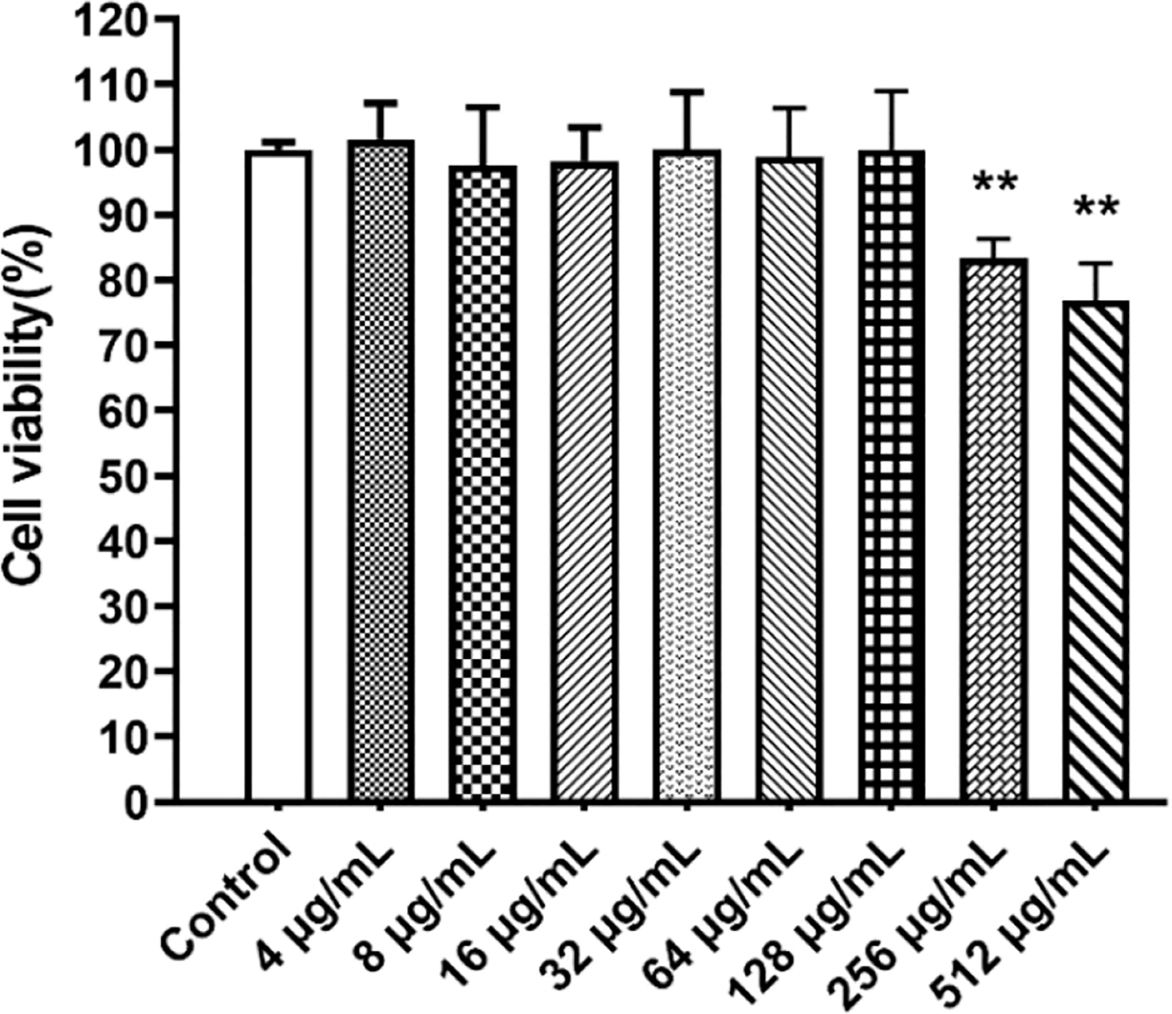
The CCK8 assay was used to determine the cytotoxicity of paeonol on RAW264.7 cells. All data were expressed as means ± SD (n=3). **P < 0.01 vs the control group.

The viability of RAW264.7 cells was performed in the presence of paeonol (0-512 µg/mL) using the CCK-8 assay. The viability of cells is more than 90% at concentrations of 0-128 µg/mL of paeonol, indicating that paeonol was not cytotoxic to RAW264.7 cells (**Figure 1**).

### The Model of Macrophages Infected With *P. aeruginosa*

**Figure 2:**
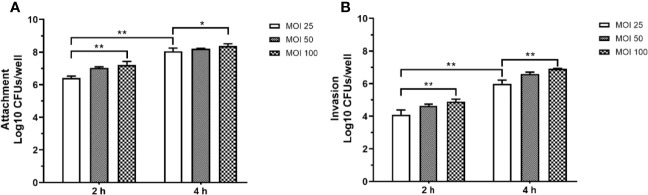
The adhesion **(A)** and invasion **(B)** of *P. aeruginosa* infected RAW264.7 cells with different MOI (10, 50, 100). The extracellular or intracellular bacteria were counted by plate. For the invasion assay, extracellular bacteria were killed with 200 μg/mL gentamicin. All data were expressed as means ± SD (n=3). *P < 0.05, **P < 0.01 vs the control group.

The prolonged *P. aeruginosa* infection has been shown to cause a significant decrease in cell viability, arrest of cell growth, and morphology alternation, ultimately weakening the host’s innate immune system’s defense function and impaired bacterial clearance function. RAW264.7 cells were infected with *P. aeruginosa* at different MOI (25, 50, 100) to explore the effect of *P. aeruginosa* infection on bacterial adhesion and invasion. **Figures 2A, B** indicated that *P. aeruginosa* could adhere to and invade the cells. As the PAO1 MOI value and infection time increased, the number of *P. aeruginosa* attaching to and invading macrophages increased (**Figure 2**).

### Paeonol Attenuate QS Genes of *P. aeruginosa* in Macrophages Infection Model

**Figure 3:**
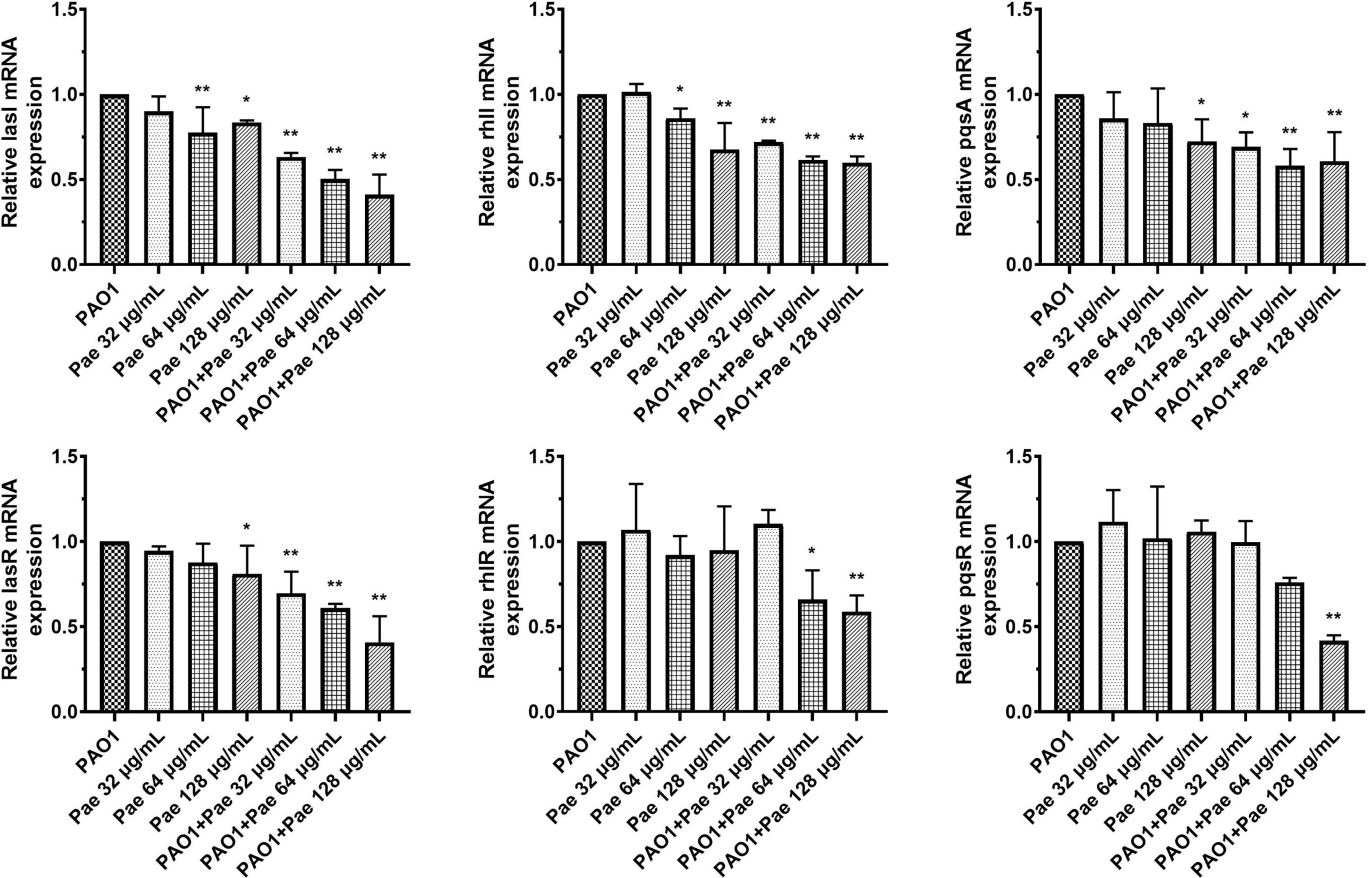
Transcriptional levels of the QS genes during *P. aeruginosa* infected macrophages (MOI = 100:1) following pretreatment and treatment with paeonol. lasI, rhlI, pqsA, lasR, rhlR, and pqsR. “Pae 32 μg/mL”, “Pae 64 μg/mL”, “Pae 128 μg/mL” indicate that RAW264.7 cells were pretreated with paeonol for 3 h before infection with PAO1. “PAO1+Pae 32 μg/mL”, “PAO1+Pae 64 μg/mL”, “PAO1+Pae 128 μg/mL” indicate that RAW264.7 cells were infected with PAO1 and treated with paeonol at the same time. The expression of mRNA was tested by RT-PCR. All data were expressed as means ± SD (n=3). *P < 0.05 or **P < 0.01 vs the control group.

*P. aeruginosa* secretes various virulence factors and forms biofilms regulated by the QS system. QS-related genes, including *lasI, lasR, rhlI, rhlR, pqsA*, and *pqsR* were analyzed following pretreatment or treatment with paeonol. The results showed that paeonol downregulated the expression of QS-related virulence genes of PAO1, and the therapeutic effect is significantly greater than the preventive effect. The inhibition rate in the presence of paeonol (128 μg/ml) was as follows: *lasI* 58.95%, *lasR* 59.20%, *rhlI* 40.19%, *rhlR* 41.20*%, pqsA* 39.33%, *pqsR* 58.26%. These results demonstrated the anti-infection activity of paeonol against *P. aeruginosa-*infected macrophages by interfering with QS-mediated related genes expression in a concentration-dependent manner (**Figure 3**).

### Paeonol Attenuate Virulence Genes During *P. aeruginosa* Infected Macrophages

**Figure 4:**
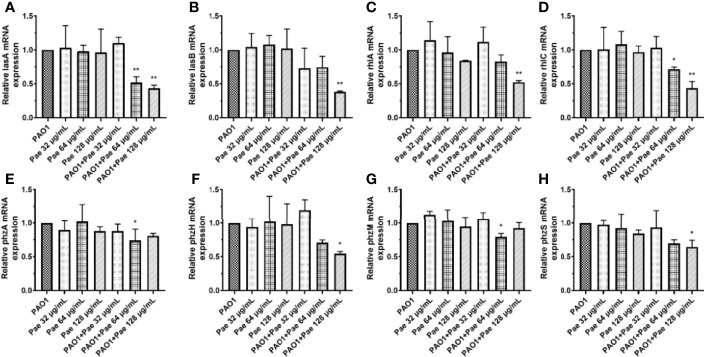
Transcriptional levels of the virulence genes during *P. aeruginosa* infected macrophages (MOI = 100:1) following pretreatment and treatment with paeonol. **(A)** lasA, **(B)** lasB, **(C)** rhlA, **(D)** rhlC **(E)** phzA, **(F)** phzH, **(G)** phzM, **(H)** phzS, “Pae 32 μg/mL”, “Pae 64 μg/mL”, “Pae 128 μg/mL” indicate that RAW264.7 cells were treated with paeonol for 3 h before infection with PAO1. “PAO1+Pae 32 μg/mL”, “PAO1+Pae 64 μg/mL”, “PAO1+Pae 128 μg/mL” indicate that RAW264.7 cells were infected with PAO1 and treated with paeonol at the same time. The expression of mRNA was tested by RT-PCR. All data were expressed as means ± SD (n=3). *P < 0.05 or **P < 0.01 vs the control group.

*P. aeruginosa* secretes a variety of virulence factors and forms biofilms, the expression of QS-regulated and QS-virulence genes was detected to assess the anti-infection activity of paeonol against *P. aeruginosa-*infected macrophages. QS system was analyzed in the macrophages cell infected *P. aeruginosa* following pretreatment and treatment at the same time with paeonol. The results showed that paeonol downregulated the expression of QS-related virulence genes in PAO1 infected RAW264.7 macrophages, and the therapeutic effect is significantly greater than the preventive effect. The inhibition rate in the presence of paeonol (128 μg/ml) was as follows: *lasA* 56.47%, *lasB* 61.81%, *rhlA* 47.65%, *rhlC* 56.74%, *phzA* 18.28%, *phzM* 7.73%, *phzH* 45.50% and *phzS* 35.47% (**Figure 4**).

### Viability and Cytotoxicity of Paeonol on Macrophages Infected With *P. aeruginosa*

**Figure 5:**
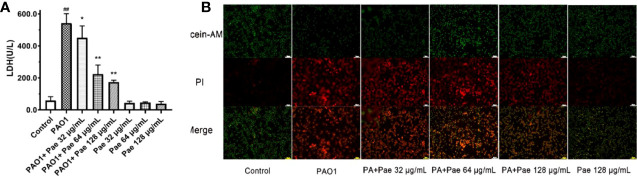
Cell viability were tested in PAO1 infected RAW264.7 (MOI = 25:1) without or with paeonol treatment for 4 h. The Fe^2+^ - xylenol orange-based LDH kit **(A)** and calcein-AM or PI dyes **(B)** were used in the testing. “Pae 32 μg/mL”, “Pae 64 μg/mL”, “Pae 128 μg/mL” indicate that RAW264.7 cells were pretreated with paeonol for 3 h before infection with PAO1. “PAO1+Pae 32 μg/mL”, “PAO1+Pae 64 μg/mL”, “PAO1+Pae 128 μg/mL” indicate that RAW264.7 cells were infected with PAO1 and treated with paeonol. ^##^p < 0.01 vs. Control Group, *P < 0.05 or **P < 0.01 vs the PAO1 group.

**Figure 6:**
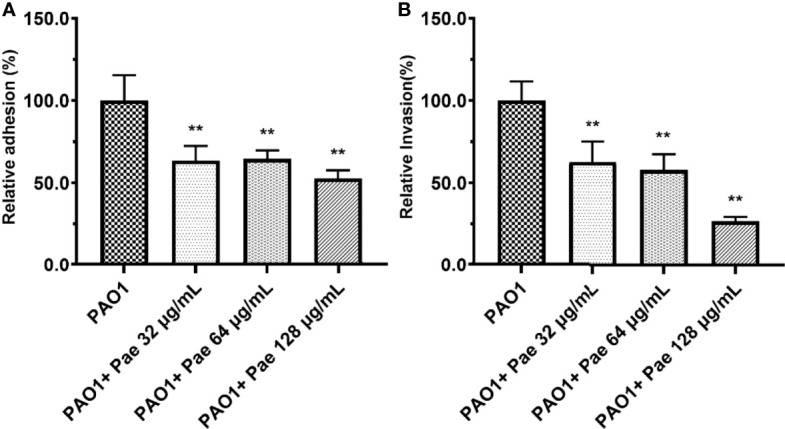
Adhesion **(A)** and invasion **(B)** were tested in PAO1-infected RAW264.7 (MOI = 25:1) with paeonol treatment for 4 hours. “PAO1+Pae 32 μg/mL”, “PAO1+Pae 64 μg/mL”, “PAO1+Pae 128 μg/mL” indicate that RAW264.7 cells were infected with PAO1 and treated with paeonol. The bacteria were cultivated and counted on a TSB plate. All data were expressed as means ± SD (n=3). **P < 0.01 vs the control group.

Bacterial infection can cause the destruction of the membrane structure of cells, resulting in the release of LDH in the cytoplasm into the culture medium. The detection of cytotoxicity can be achieved by detecting the activity of LDH in the culture medium. The LDH levels were detected by Fe^2+^ - xylenol orange-based kit. Compared with the control (RAW264.7), PAO1 significantly inhibited the proliferation of macrophages cells, but the release of LDH decreased in a concentration-dependent manner by paeonol treatment, and paeonol did not affect the proliferation of macrophages at the experimental concentrations (**Figure 5A**). To directly examine the cell viability or cytotoxicity, RAW264.7 cells were exposed to PAO1 treated without or with paeonol, then labeled with Calcein-AM and PI dyes and immediately observed by fluorescence microscope. **Figure 6** illustrates that macrophages cells infected with PAO1 were mainly dead (red, propidium iodide-positive), but treated with paeonol groups were mainly viable cells (green, calcein-positive) and a few dead cells. The results indicated that paeonol within 128 μg/mL is not cytotoxic to RAW264.7 cells (**Figure 5B**).

### Paeonol Decreased the Adhesion and Invasion of PAO1

RAW264.7 cells were infected with PAO1 to determine the bacterial adhesion and invasion. Compared with the PAO1 group, bacteria adhesion on macrophages significantly decreased after paeonol treatment. Further study revealed that paeonol reduced the invasion of PAO1 to RAW264.7 cells (**Figure 6**).

### Effects of Paeonol on the Ultrastructure of RAW264.7 Macrophages Infected by PAO1

**Figure 7:**
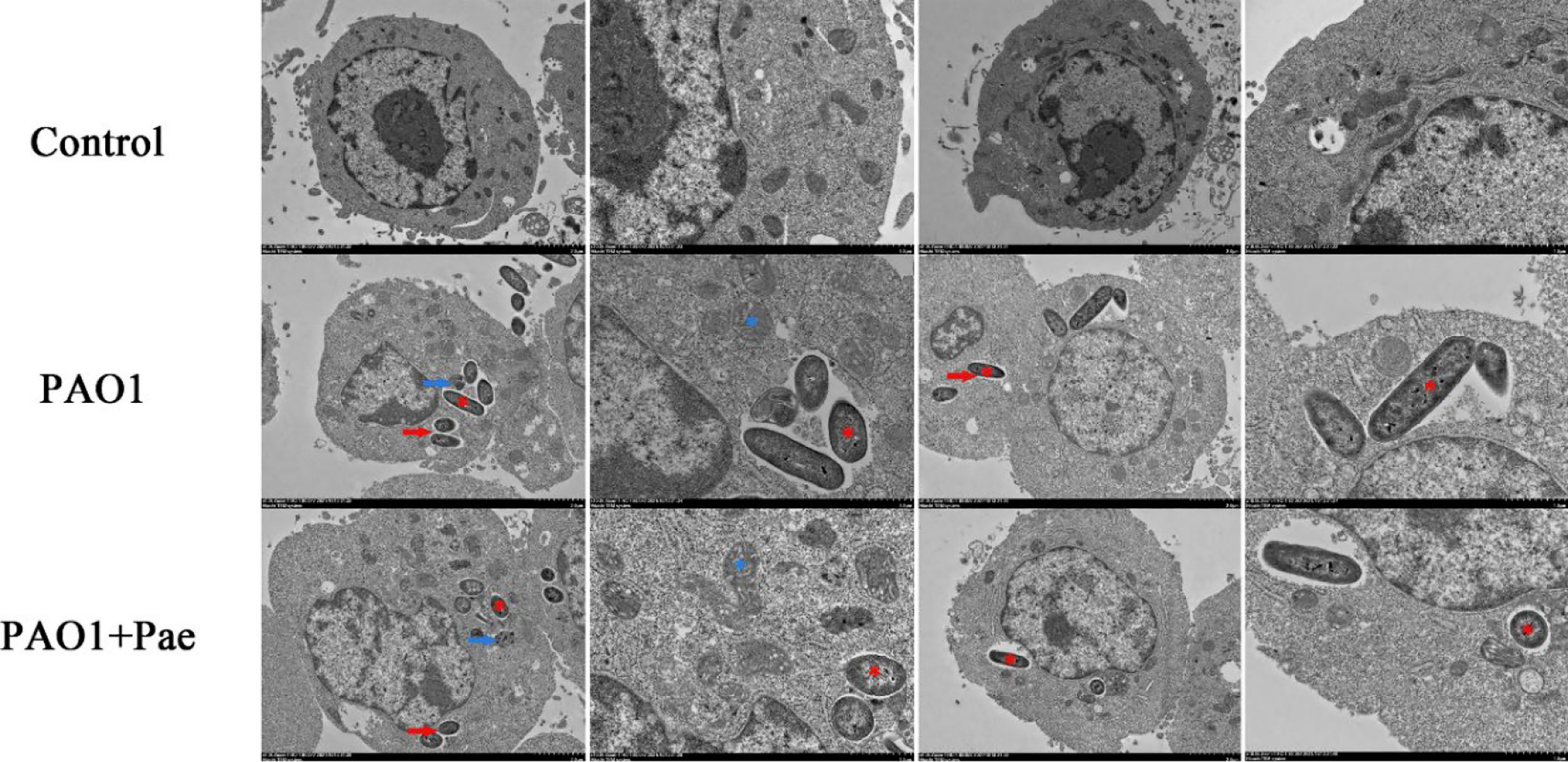
Transmission electron microscope observation of paeonol on the ultrastructure of RAW264.7 macrophages infected with *P. aeruginosa* PAO1 (MOI=25:1). Scale bar, 2 μm (low magnification in columns one and three) and 1 μm (high magnification in columns two and four); red* indicates PAO1, blue* indicates mitochondria; red arrows indicate vesicles, blue arrows indicate autophagosome-lysosomes.

TEM was performed to detect the ultrastructure of RAW264.7 macrophages infected by PAO1 and treated with paeonol. As shown in **Figure 7**, there were no significant changes in the blank control group. In the PAO1 group, *P. aeruginosa* invaded macrophages, PAO1 was encapsulated by vesicles, part of the vesicle membrane was destroyed, mitochondria were swollen and vacuolized, escaped phagosomes into the cytoplasm, causing nuclear shrinkage, and even cell damage or lysis. In the paeonol(128 μg/mL) treated group, *P. aeruginosa* invasion was reduced and encapsulated by intact vesicles, the mycelium changed from rod-like to round, the fusion between PAO1 and lysosome increased, the mitochondria slightly swollen and more filamentous, the nucleus of the cell was intact, and the cell injury was reduced.

### Paeonol Suppressed Inflammation in RAW264.7 Cells Infected *P. aeruginosa*

**Figure 8:**
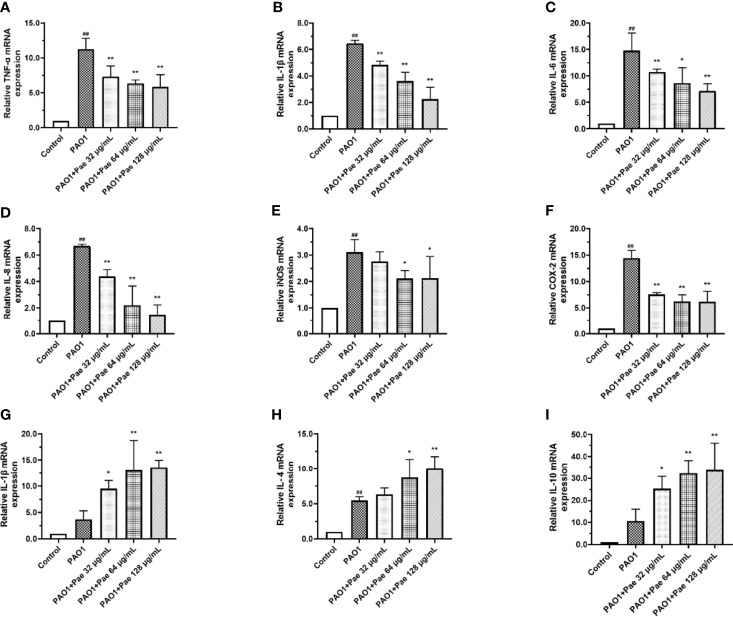
Cytokines mRNA expression were tested in PAO1-infected RAW264.7 (MOI = 100:1) with paeonol treatment. **(A)** TNF-α, **(B)** IL-1β, **(C)** IL-6, **(D)** IL-8, **(E)** iNOS, **(F)** COX-2, **(G)** IL-2, **(H)** IL-4, **(I)** IL-10, “PAO1+Pae 32 μg/mL”, “PAO1+Pae 64 μg/mL”, “PAO1+Pae 128 μg/mL” indicate that RAW264.7 cells were infected with PAO1 and treated with paeonol. The expression of mRNA was tested by RT-PCR. All data were expressed as means ± SD (n=3). ^#^p < 0.05, ^##^p < 0.01 vs. control Group, *P < 0.05 or **P < 0.01 *vs* the PAO1 group.

Compared with the control group, the relative mRNA expression level of IL-1β, IL-6, IL-8, TNF-α, COX2, and iNOS in the PAO1 group was upregulated, respectively. Compared with the PAO1 group, paeonol can attenuate the expression of inflammatory cytokines like IL-1β, IL-6, IL-8, TNF-α, various inflammatory mediators such as COX2, iNOS, and upregulated the relative mRNA expression level of IL-2, IL-4, IL-10. Paeonol treatment significantly prevented the inflammation changes induced by *P. aeruginosa* in a dose-dependent manner (**Figure 8**).

### Paeonol Changed the Polarization of Macrophages

**Figure 9:**
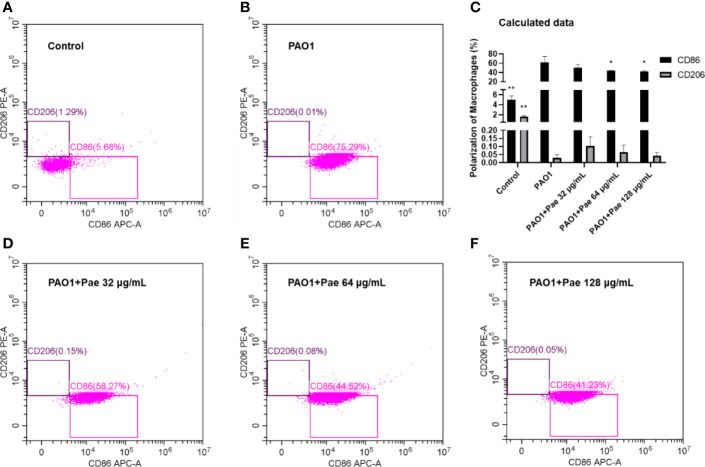
Paeonol changed the Polarization of Macrophages infected with PAO1 (MOI = 25:1). Control **(A)**, PAO1 **(B)** The data in “PAO1+Pae 32 μg/mL” **(D)**, “PAO1+Pae 64 μg/mL” **(E)**, and “PAO1+Pae 128 μg/mL” **(F)** indicated that Paeonol reversed the upregulated the biomarker of CD86 of RAW264.7 cells infected with PAO1 **(C)**. The cell polarization was distinguished by Flow cytometry. All data were expressed as means ± SD (n=3). *P < 0.05 or **P < 0.01 *vs* PAO1 group.

Flow cytometry was used to detect macrophage polarization. F4/80 biomarker was used to label macrophages, and CD86/CD206 anti-body was used to check the surface biomarker of macrophages, respectively. The CD86 biomarker significantly was significantly upregulated when macrophages were infected with PAO1. Paeonol significantly decreased the polarization of macrophages by decreeing the CD86 biomarker when the treatment concentrations higher than 64 μg/mL (**Figure 9**).

### Effect of Paeonol on the TLR4/MyD88/NF-κB Pathway of Macrophages Infected With *P. aeruginosa*

**Figure 10:**
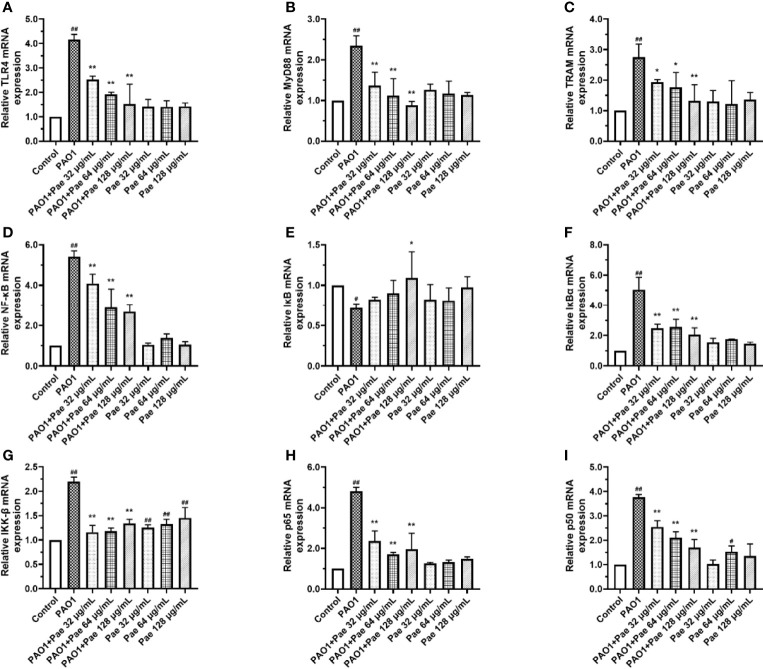
Paeonol inhibited the activation of TLR4/MyD88/NF-κB pathway in PAO1 infected RAW264.7 (MOI = 100:1). RAW264.7 were exposed to PAO1 treated without or with paeonol for 4 h to determine the mRNA expression levels of the TLR4/MyD88/NF-κB pathway. **(A)** TLR4, **(B)** MyD88, **(C)** TRAM, **(D)** NF-κB, **(E)** IκB, **(F)** IκBα, **(G)** IKK-β, **(H)** p65, **(I)** p50, “PAO1+Pae 32 μg/mL”, “PAO1+Pae 64 μg/mL”, “PAO1+Pae 128 μg/mL” indicate that RAW264.7 cells were infected with PAO1 and treated with paeonol. “Pae 32 μg/mL”, “Pae 64 μg/mL”, “Pae 128 μg/mL” indicate that RAW264.7 cells were treated with paeonol but without PAO1 infection. The expression of mRNA was tested by RT-PCR. All data were expressed as means ± SD (n=3). ^#^p < 0.05, ^##^p < 0.01 vs. Control Group, *P < 0.05 or **P < 0.01 vs the PAO1 group.

TLR4/MyD88/NF-κB pathway is the fetal inflammation pathway for macrophages responding to the bacteria infection. To investigate the effect of paeonol on the TLR4/MyD88/NF-κB signaling pathway, the mRNA expression levels of TLR4, MyD88, TRAM, NF-κB, IκB, IκBα, IKK-β, p65, and p50 were measured by qPCR. In Paeonol treated cells, the mRNA expression levels of TLR4, MyD88, TRAM, NF-κB, IκB, IκBα, IKK-β, p65, and p50 were significantly downregulated, and IκB was markedly higher in comparison with those in the PAO1 group. These results demonstrated that paeonol attenuated *P. aeruginosa*-induced inflammation as evidenced by decreased expressions of TLR4/MyD88/NF-κB pathway in RAW264.7 cells, data were included in **Figure 10**.

### Paeonol Showed an Effective Anti-Infection Activity *In Vivo*

**Figure 11:**
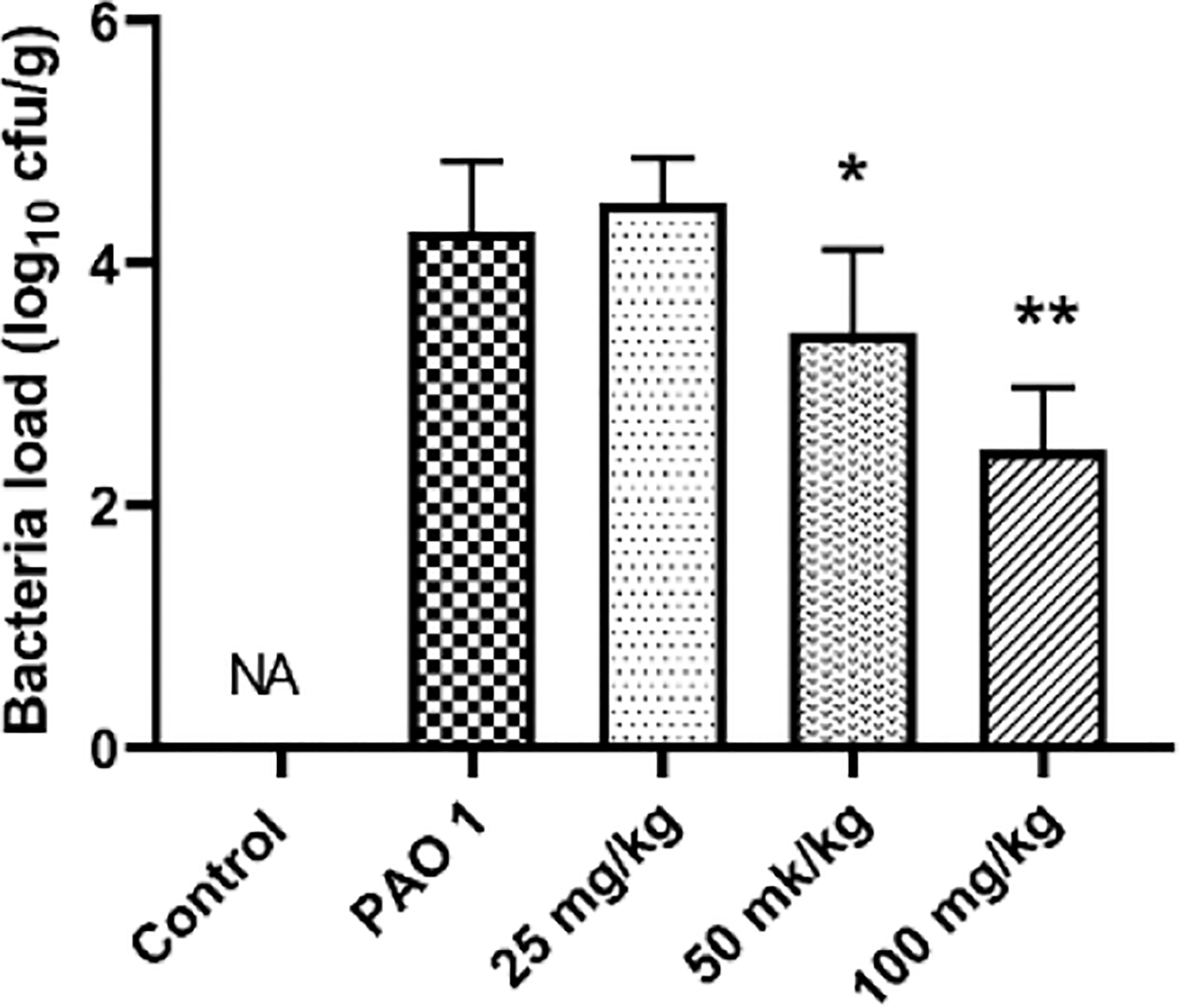
Paeonol decreased the bacterial load in the lung. All data were expressed as means ± SD (n=3). *P < 0.05 or **P < 0.01 vs PAO1 group.

**Figure 12:**
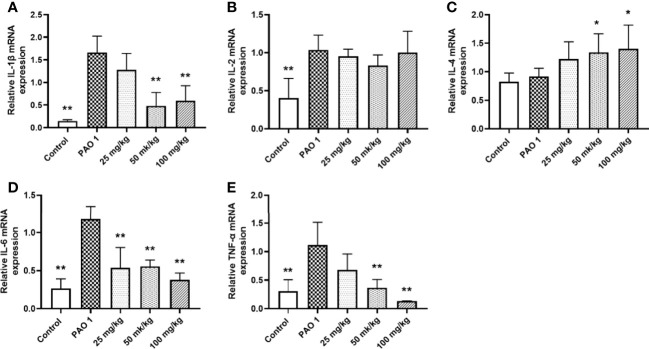
Paeonol inhibited the mRNA expression of inflammation cytokines in the lung. All data were expressed as means ± SD (n=3). **(A)** IL-1β, **(B)** IL-2, **(C)** IL-4, **(D)** IL-6, **(E)**TNF-α. *P < 0.05 or **P < 0.01 vs PAO1 group.

**Figure 13:**
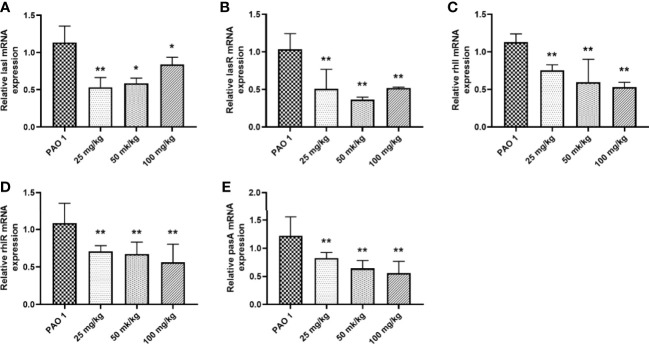
Paeonol inhibited the mRNA expression of QS genes in the lung. **(A)** lasI, **(B)** lasR, **(C)** rhlI, **(D)** rhlR, **(E)** pqsA. All data were expressed as means ± SD (n=3). *P < 0.05 or **P < 0.01 vs PAO1 group.

Paeonol decreased the PAO1 load in the lung and inhibited the mRNA expression of inflammation cytokines, including IL-1β, IL-6, and TNF-α (**Figures 11**, **12**). The mRNA expression of IL-4 was increased by IL-4. All three concentrations of paeonol decreased the expression of quorum sensing-related genes, *rhlR*, *LasR*, and *pqsA* (**Figure 13**).

## Discussion

Due to the excessive and indiscriminate use of antibiotics, multidrug-resistant (MDR) bacteria are rapidly increasing and difficult to control by using conventional antibiotics (33–35). *P. aeruginosa*, a prevalent nosocomial-acquired and opportunistic pathogen, causes various acute and chronic infections, often involving the synergy of multiple virulence gene regulatory factors (36, 37). Macrophages are crucial innate immune cells that play a critical role in the host’s response to bacterial infection. Macrophages pattern recognition receptors (PRRs), especially TLRs, recognize pathogen-associated molecular patterns (PAMPs) and activate intracellular various signaling pathways, such as inflammation, phagocytosis, apoptosis, and autophagy (38). Excessive inflammatory cytokines are detrimental to the host at the later stages of infection, and limiting excessive inflammatory response is crucial to controlling *P. aeruginosa* infection (39).

In the model of macrophages infected with *P. aeruginosa*, there was a significant up-regulation in the number of bacterial attachments and invasions to cells as the MOI value and infection time increased (40, 41). Therefore, the increase of co-aggregation between *P. aeruginosa* promotes the invasion of bacteria to macrophages, which may enable bacteria to evade host immune defense and cause great damage to host cells. The pathogenesis of *P. aeruginosa* is closely associated with biofilm formation, motility, and a myriad of extracellular virulence factors regulated by the QS system (42, 43). Large-scale expression of QS-related signaling molecules (AHL) or virulence factors (pyocyanin, protease, elastase, and rhamnolipids) were cytotoxic to macrophages (44). PAO1 significantly inhibited macrophages cell proliferation, induced cytotoxic responses, and led to cell membrane damage and rupture, lysis, and death. Our former studies had revealed that paeonol could attenuate quorum-sensing regulated virulence and biofilm formation in *P. aeruginosa* and show a protective effect on *C. elegans* infected with *P. aeruginosa in vivo* (28). Here, the potential therapeutic effect and mechanism of paeonol were investigated in a macrophage infected with a PAO1 cell model. The results revealed that paeonol inhibited *P. aeruginosa* QS-signaling pathway (lasI/R, rhlI/R, pqsA/pqsR) and virulence factors (*lasA, lasB, rhlA, rhlC, phzA, phzM, phzH, and phzS*) to alleviate the PAO1 induced cell damage (28).

The attachment and invasion of host cells is the key step in the *P. aeruginosa* infection cycle, and macrophages employ a variety of defense mechanisms to defend against invading pathogens. Phagocytosis and intracellular killing are two of the most important steps for bacterial eradication (45, 46). The motility of *P. aeruginosa* regulated by the QS system was crucial for colonization (13, 47). Studies showed that paeonol could resist the adhesion and invasion of *P. aeruginosa* to infect macrophages, which may be related to the fact that paeonol reduced the motility ability of *P. aeruginosa*. Previous studies had shown that *P. aeruginosa* could directly induce cell death to aggravate tissue damage through its QS system or toxic factors such as pyocyanin (48, 49). TEM results showed that PAO1 co-polymerized with each other to adhere to cell junctions and invaded the interior of cells. Compared with the PAO1 group, the phagocytosis ability of macrophages was enhanced in the paeonol treatment groups. Paeonol resists the cell toxicity induced by *P. aeruginosa* and improves cell viability. This work confirmed the effect of paeonol against *P. aeruginosa* by attenuating virulence factors and its cytoprotective activity during bacterial infection either by downregulating the virulence or providing a protective effect to the host cells. Zhang et al. have reported that phagocytic macrophages undergo apoptosis 48 h after ingestion of *P. aeruginosa* (50). Moussouni et al. have reported that OprF regulates *P. aeruginosa* virulence factors and protects macrophages during acute infection by avoiding phagolysosome destruction (51).

It has been reported that *P. aeruginosa* infection often exacerbates symptoms in patients, leading to organ dysfunction, followed by systemic inflammatory response syndrome and increased morbidity and mortality (52, 53). As an indispensable part of innate immunity, macrophages play an important role in inflammatory and immune responses. In this study, paeonol treatment significantly reduced *P. aeruginosa*-induced proinflammatory cytokine and improved anti-inflammatory factors produced by macrophages. The anti-inflammatory effect of paeonol was demonstrated in a PAO1-infected macrophage model and a PAO1 infected pneumonia model, and the results were consistent with LPS-induced RAW264.7 inflammatory factors (30). Generally, activated macrophages differentiate into M1 or M2 phenotypes. M1 macrophages (the classically activated, proinflammatory) increase the level of oxidative stress-induced products, and tissue damage was noted in the acute inflammatory phase, and M2 macrophages (the alternative activated, anti-inflammatory) decrease inflammation and promote tissue repair (54, 55). Classically activated (M1) macrophages can be triggered by recognizing Gram-negative bacteria, and cascade inflammatory responses will be triggered. TLR4 recruits myeloid MyD88, leading to proinflammatory cytokine production with activation of NF-κB and the downstream gene targets (56, 57). The expression of key genes in the TLR4/MyD88/NF-κB signaling pathway and proinflammatory cytokines were induced by *P. aeruginosa* infection. Atter treatment with paeonol, the activation of inflammation pathway, expression of proinflammatory cytokines, and the M1 macrophages polarization were significantly inhibited in our results. Paeonol against PAO1 infection by inhibiting bacterial virulence and enhancing the clearance of pathogen by immune cells, which avoided severe inflammatory damage to cells and body. Collectively, this suggests that paeonol may represent a new promising anti-QS and anti-inflammatory agent that may prevent *P. aeruginosa*-mediated impairment of inflammation and infection.

## Conclusion

This research exhibited direct evidence that paeonol possessed the anti-QS activity against *P. aeruginosa* virulence, which decreased the adhesion, invasion, and cytotoxicity of *P. aeruginosa* to macrophages. Paeonol improved cell viability in the cell model of macrophages infected with *P. aeruginosa* by inhibiting the expression of QS-related virulence genes of *P. aeruginosa* and reducing the activation of macrophage proinflammation cytokines and inflammation pathway. Paeonol also decreased the bacterial load and alleviated the inflammation response in a *P. aeruginosa* pneumonia model.

## Data Availability Statement

The raw data supporting the conclusions of this article will be made available by the authors, without undue reservation.

## Ethics Statement

The animal study was reviewed and approved by Sichuan Agricultural University.

## Author Contributions

HT and DY designed and performed the study, analyzed the data, and wrote the draft paper. LZhu, FS, GY, RG, HD, and LZhao performed experiments, analyzed the data, and edited the manuscript. ZW and YL designed, organized, supervised research, and edited the paper. All authors contributed to the paper and approved the submitted version.

## Funding

This study was supported by the International Science and Technology Cooperation Program of Sichuan: 2020YFH0143.

## Conflict of Interest

The authors declare that the research was conducted in the absence of any commercial or financial relationships that could be construed as a potential conflict of interest.

## Publisher’s Note

All claims expressed in this article are solely those of the authors and do not necessarily represent those of their affiliated organizations, or those of the publisher, the editors and the reviewers. Any product that may be evaluated in this article, or claim that may be made by its manufacturer, is not guaranteed or endorsed by the publisher.
